# Patient-Specific Therapy via Cell-Reprogramming Technology: a Curative Potential for Patients with Diabetes

**DOI:** 10.1186/s11671-015-1193-8

**Published:** 2015-12-29

**Authors:** Haizhao Luo, Xianbao Wang, Ruyi Zhang, Youping Chen, Yi Shu, Huixian Li, Hong Chen

**Affiliations:** Department of Endocrinology, Nanhai Hospital, Southern Medical University, No. 40, Fo Ping Road, Foshan, 528200 China; Department of Cardiology, Zhujiang Hospital, Southern Medical University, No. 253, Gong Ye Road, Guangzhou, 510282 China; Department of Endocrinology, Guangzhou Red Cross Hospital, No. 396, Tongfu Zhong Road, Guangzhou, 510220 China; Department of Endocrinology, Zhujiang Hospital, Southern Medical University, No. 253, Gong Ye Road, Guangzhou, 510282 China

**Keywords:** Gene therapeutics, Ultrasound, Microbubbles, Cross-linked polyethylenimines

## Abstract

Gene therapeutics provides great opportunities for curing diabetes. Numerous attempts have been made to establish a safe and high-efficiency gene delivery strategy, but all of them are unsuccessful. To achieve an ideal transfection, a novel gene delivery strategy was presented in this research. The novel system proposed was transfection mediated by the combination of ultrasound with microbubbles and cross-linked polyethylenimines (PEIs). Ultrasound with microbubbles enhances the permeability of target cells; moreover, cross-linked PEIs enabled DNA to escape from endosomes into the cytoplasm. If the proposed method is feasible and effective, the endogenous secretion system of insulin would be re-established in patients with diabetes.

## Background

Currently, diabetes mellitus has become a severe global public healthcare problem due to the increasing prevalence, morbidity, and mortality. Islet transplantation has become a promising strategy for curing diabetes following the success of the “Edmonton protocol” [1]. However, islet transplantation is limited by both scarcity of donor cells and requirement for life-long immunosuppressive therapy. Gene therapeutics has been heavily investigated to be a new treatment for this disease. The most pivotal task is to achieve a clinically safe and efficient gene delivery method.

**Fig. 1:**
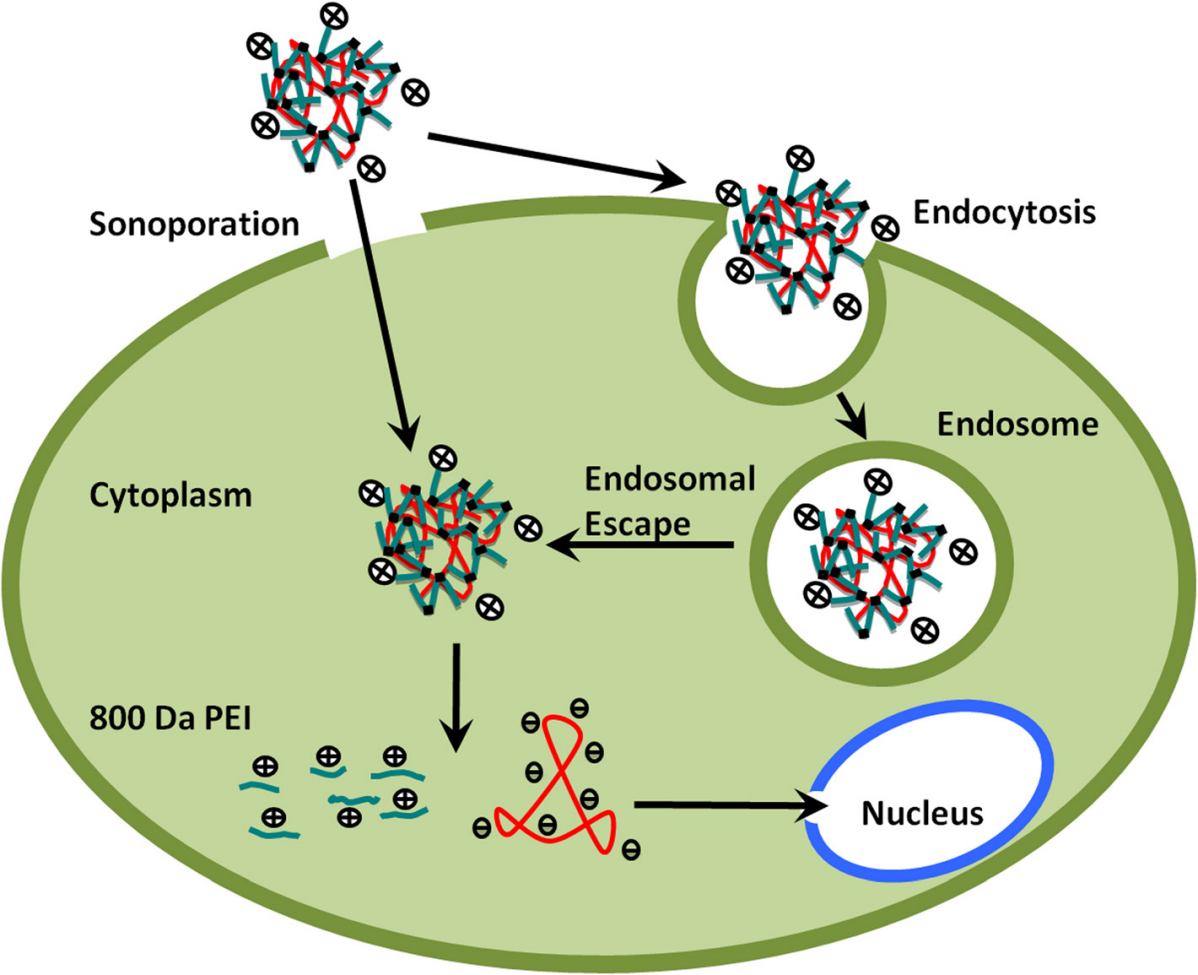
Schematic drawing of gene transfection by the combination of US with microbubbles and cross-linked PEIs. On one hand, sonoporation of US allows cross-linked PEI-DNA complexes into the cytoplasm. On the other hand, US can enhance endocytosis. The complexes will escape from the endosome to the cytoplasm. After reaching the cytoplasm, all the complexes will degrade into their low molecular-weight components and release DNA to enter the nucleus for transcription

**Fig. 2:**
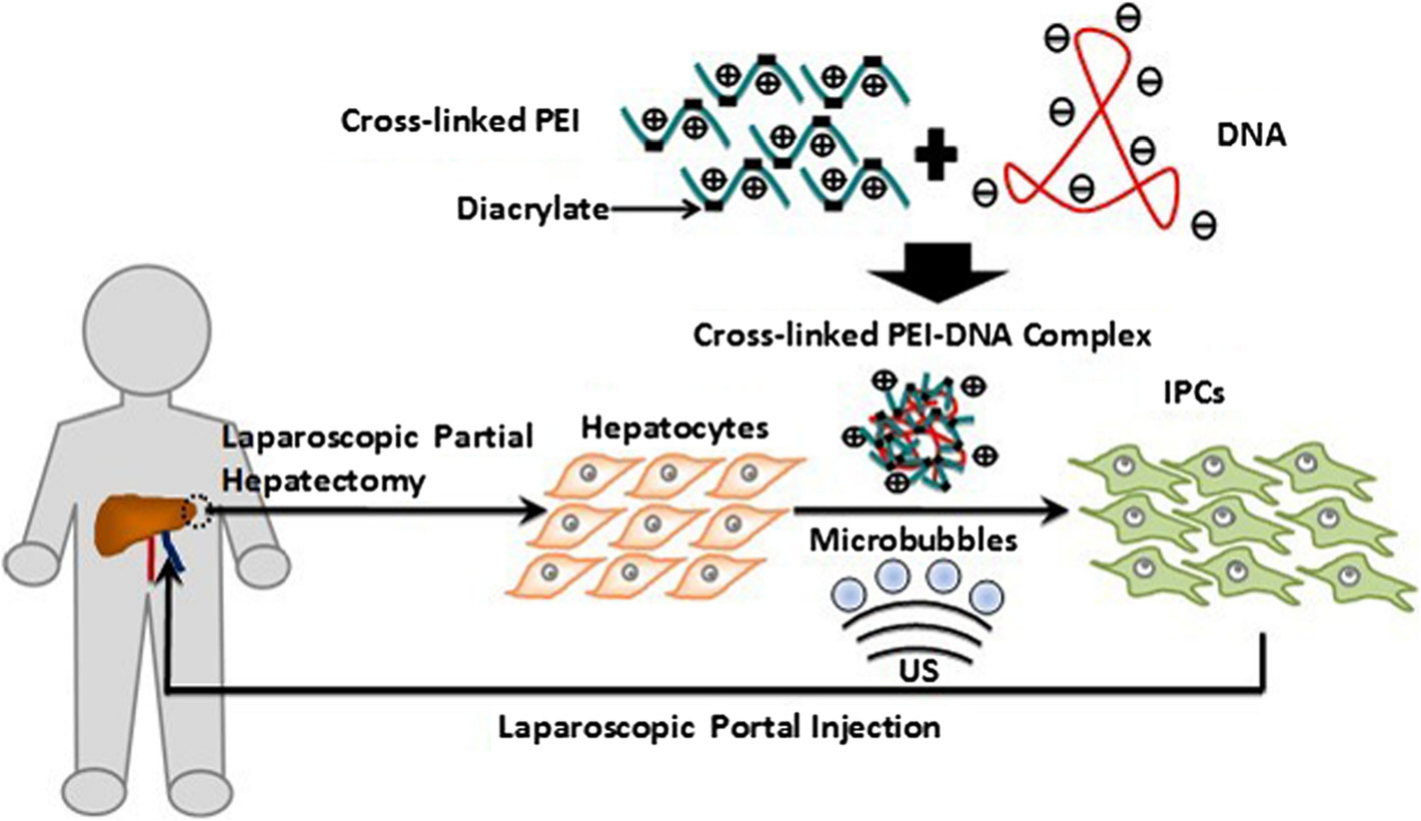
Scheme of the patient-specific therapy using cell-reprogramming technology. Hepatocytes are obtained from a diabetic patient by a laparoscopic partial hepatectomy. Cross-linked polyethylenimine (PEI)-DNA complexes are transfected into these hepatocytes with the aid of ultrasound (US) with microbubbles. The transfection leads to trans-differentiation from hepatocytes into insulin-producing cells (IPCs). The induced IPCs are auto-transplanted to the liver via laparoscopic portal injection, to re-establish the endogenous secretion system of insulin

In the last two decades, transgenic system based on viral vectors held promises for gene delivery for the efficient delivery and long-term expression of transgenes to target cells [2]. It was documented that viral vectors-mediated transgenes in hepatocytes could ameliorate hyperglycemia in mice [3, 4]. By 2012, viral vectors have been employed as gene carriers in most of researches and clinical trials [5]. However, safety concerns in gene delivery have been highly controversial. Theoretically, viral vectors would induce unexpected immune response and oncogenic effects. In fact, a tragic gene therapy death of a volunteer in a clinical study was caused by administering a high dosage of an adenovirus vector within 98 h [6]. The post-mortem examination revealed that the patient died of multi-organ failure due to the fatal immune response triggered by the administered adenovirus [7].

Non-viral approaches have also been extensively investigated as alternatives over past a few years. Compared to viral methods, they are likely to present fewer toxic and immunological problems. However, inefficient gene transfer was still their “Achilles heel” currently [8]. It is believed that when only a new non-viral method is explored, the large-scale clinical application of gene therapy would be allowed.

## Presentation of the Hypothesis

Given the need, a novel therapeutic strategy using ultrasound (US) with microbubbles technology has entered the realm of gene delivery. Firstly, microbubbles, encapsulating gas in micron-sized shell, were introduced as US contrast agent to improve the ultrasonic imaging [9]. Recent investigations suggested that US with the aid of microbubbles is a potential novel method for the gene therapy in various diseases, both in vivo [10, 11] and in vitro studies [12, 13]. It was addressed that gas-filled microbubbles were driven in US field, and induced shear stresses and sudden ruptures, which subsequently generated force and then punctured on the surrounding cells and tissues, which was termed as sonoporation [14]. Finally, reversible pores resulted from the force enhanced the permeability of the cell membrane temporarily and permitted foreign genes into cells [12, 15, 16]. Besides, endocytosis stimulated by US is another reason for the facilitation of this gene delivery system [17]. Compared with viral methods, many desirable features of gene therapy such as safety, low cytotoxicity, low immunogenicity, and low cost can be found in the gene delivery strategy of US with microbubbles. Whereas this protocol still suffers from the drawback of insufficient transfection efficiency [18, 19].

Cross-linked polyethylenimine (PEI) is a vector tailor-made for gene delivery, which was synthesized by adding 800-Da PEIs to small diacrylate cross-linkers. The degradable polymers exhibited characteristics of similar in structure, size, and DNA-binding properties to off-the-shelf 25-kDa PEI. Notably, they are essentially non-toxic for high efficiencies [20]. Compact nanoparticles are formed through electrostatic attraction between negative charge DNA and positive charge cross-linked PEIs. These particles can escape from the endosomes into the cytoplasm, and prevent DNA from being digested [21]. After reaching the cytoplasm, the particles will decompose into their low molecular-weight components, which are believed to be essentially non-toxic, and then release DNA to enter the nucleus for transcription. Moreover, the synergistic effect of transfection efficiency in combination of US with microbubbles and 25-kDa PEIs was found in Tu and his colleagues’ study [22]. Therefore, we believe that greater transfection efficiency might be achieved via the combination of US with microbubbles and cross-linked PEIs (Fig. 1).

## Implications of the Hypothesis

It was proved that transfection with Pdx1, Ngn3, and MafA was a most efficient combination in reprogramming hepatocytes into insulin-producing cells that closely resemble endogenous $\beta$-cells in our previous study [23]. It is expected that a safer and more efficient transfection can be achieved by our proposed gene delivery strategy. Laparoscopic approach has been confirmed as a reliable therapeutic option in hepatectomy. Therefore, it is also expected that diabetes could be cured by their own reprogrammed hepatocytes via introducing Pdx1, Ngn3, and MafA (Fig. 2).
